# Prevalence of Sarcopenia and Its Association with Nutrition Risk in Community-Dwelling Older Adults with Subjective Cognitive Impairment or Living in Retirement Homes

**DOI:** 10.3390/nu18162733

**Published:** 2026-08-21

**Authors:** Raksha Aravind, Heather H. Keller

**Affiliations:** 1 Kinesiology and Health Sciences, University of Waterloo, 200 University Ave W, Waterloo, ON N2L 3G1, Canada; r4aravind@uwaterloo.ca; 2 Schlegel-UW Research Institute for Aging, 250 Laurelwood Dr., Waterloo, ON N2J 0E2, Canada

**Keywords:** sarcopenia, nutrition risk, SCREEN-14, older adults, retirement homes, subjective cognitive impairment, EWGSOP2, malnutrition

## Abstract

Background: Sarcopenia is an age-related condition characterized by declines in muscle strength and muscle mass associated with adverse health outcomes in older adults. Nutrition risk may contribute to sarcopenia development; however, its relationship with sarcopenia among community-dwelling older adults, especially those living in retirement homes or with subjective cognitive impairment, remains unclear. This study aimed to determine the prevalence of sarcopenia and its indicators using the EWGSOP2 criteria in community-dwelling older adults with cognitive impairment and those living in retirement homes and examine associations between nutrition risk and probable sarcopenia. Methods: A secondary analysis of cross-sectional data from 326 adults aged ≥55 years was conducted. Sarcopenia was assessed using the EWGSOP2 Find–Assess–Confirm–Severity framework and nutrition risk was measured using SCREEN-14 and the Nutrition Risk Rating Score (NRRS). Results: Overall, 37.0% of participants were at risk for sarcopenia, and 26.9%, 5.5%, and 4.3% were classified with probable, confirmed, and severe sarcopenia, respectively. Retirement home residents were more likely to meet criteria for sarcopenia risk, low grip strength, and slow gait speed (all *p* < 0.001) than those with subjective cognitive impairment living in the community. In adjusted analyses, walker use and higher NRRS were associated with probable sarcopenia, whereas SCREEN-14 scores were not. Conclusions: These findings suggest that sarcopenia risk is common in these populations but confirmed sarcopenia is low using the EWGSOP2 framework. Nutrition risk as assessed by a dietitian using a comprehensive assessment is associated with probable sarcopenia.

## 1. Introduction

Sarcopenia is a progressive, age-related condition marked by the decline of skeletal muscle mass and muscle strength [1,2]. It leads to increased risks of physical disabilities, falls, fractures, dependency, and mortality, affecting both quality of life and healthcare needs across various settings [3,4,5,6,7]. Estimates suggest that sarcopenia affects 10–40% of older adults worldwide, highlighting its widespread impact [8]. Additionally, the global burden of sarcopenia is expected to rise, with projections suggesting that the number of affected individuals could reach approximately 1.2 billion by 2025 and double this number by 2050 [3]. Based on recent data, the proportion of Canadians aged 65 and over was 19% in 2023, with estimates indicating older adults will comprise between 22% and 32% of the population by 2073 [9]. The projected growth of the aging Canadian and worldwide population is expected to increase the number of individuals affected by sarcopenia, reinforcing the need for early identification and intervention.

**Figure 1:**
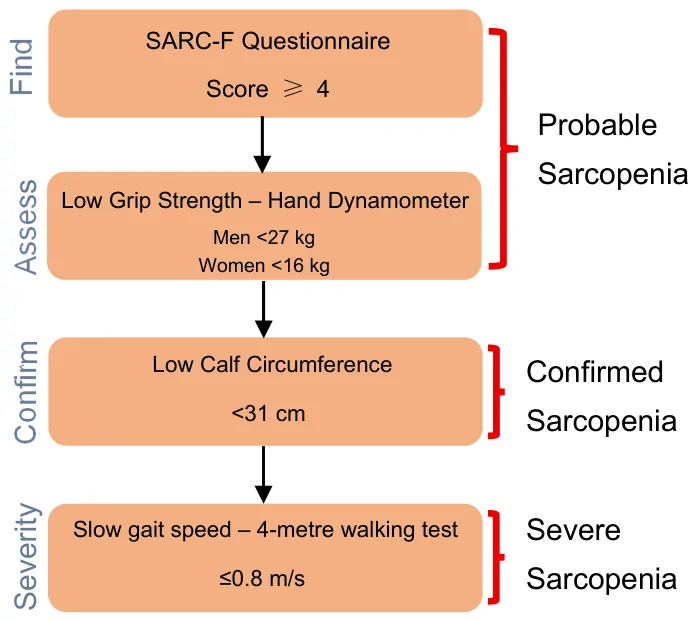
EWGSOP2 criteria for sarcopenia diagnosis.

With the increasing recognition of sarcopenia, its definitions and measurements have been in flux. Several expert groups have proposed operational definitions, examples including the European Working Group on Sarcopenia in Older People (EWGSOP/EWGSOP2), the Asian Working Group for Sarcopenia (AWGS), the Foundation for the National Institutes of Health (FNIH), the International Working Group on Sarcopenia (IWGS), the European Society of Clinical Nutrition and Metabolism (ESPEN), and the International Sarcopenia Initiative [10]. Differences in the components included, recommended assessment methods, and diagnostic cut-points have contributed to variation in sarcopenia prevalence estimates and limited comparability across studies [10]. Recently, the Global Leadership Initiative in Sarcopenia (GLIS) established the first international conceptual definition of sarcopenia through a global Delphi consensus. GLIS has noted that the next step is an operational definition including establishing the specific measurement methods and diagnostic thresholds [2]. Therefore, this study used the EWGSOP2 operational framework (Find–Assess–Confirm–Severity (F-A-C-S) approach), which remains one of the most widely accepted and validated approaches for diagnosing sarcopenia in research settings [2,11]. Figure 1 illustrates the EWGSOP2 diagnostic pathway. Details of the measures and cut-off points used in this study are described in the Methods section.

According to the ESPEN guidelines and the Global Leadership Initiative on Malnutrition (GLIM), malnutrition is a condition resulting from inadequate nutrient intake or absorption that leads to altered body composition, including reductions in fat-free mass (FFM) or skeletal muscle mass, alongside impaired physical and mental function and adverse clinical outcomes [1,12]. The assessment of FFM or muscle mass forms a key component of both GLIM-defined malnutrition and sarcopenia assessment [1]. Sarcopenia is distinguished by impaired muscle strength and physical performance, whereas malnutrition is defined by the combination of phenotypic and etiologic criteria [1]. Consequently, malnutrition and sarcopenia often coexist because of shared underlying mechanisms, including muscle loss, inadequate nutritional intake, inflammation, and functional decline [13]. Both conditions can manifest through weight loss, diminished strength, and reduced physical capacity, creating a cycle of decreased activity, frailty, and further muscle deterioration [13].

Risk of malnutrition is a dynamic state that precedes and can lead to malnutrition [14]. Weight loss and risk factors (nutrition impact symptoms, physical, psychological, social, demographic or economic) are common [14]. This definition of risk of malnutrition is considered relevant for all healthcare settings. For this work, nutrition risk is conceptualized as a state preceding malnutrition and potentially risk of malnutrition, depending on how it is measured, for those specifically living in the community [15]; in this context, weight change, gain or loss, are both considered as well as self-report of food and fluid intake and risk factors that impair food and fluid intake [16].

Identification of nutrition risk and risk of sarcopenia remains limited in non-clinical populations, such as older adults living in retirement homes and community-dwelling older adults with subjective cognitive impairment. Older adults in Canada choose retirement homes as a residential option that provides meals and housekeeping [17]. Healthcare is limited to home care provided by the regional government or purchased services provided by the home (e.g., medication management) [17]. It has been recognized that this group of older adults are often more similar to nursing home residents who require twenty-four-hour nursing care and thus could be at increased risk for malnutrition and sarcopenia [17,18]. Similarly, older adults with subjective cognitive impairments or dementia may have instrumental and basic activities of daily living affected, leading to decreased function, malnutrition risk and sarcopenia [19,20]. Malnutrition has been demonstrated to be higher in these individuals through a variety of mechanisms that impact food intake [21,22,23].

This study had the following objectives: (1) to determine the difference in prevalence of sarcopenia and sarcopenia indicators as per EWGSOP2 in persons living in Ontario retirement homes and older adults living in the community with subjective cognitive impairment, and (2) to examine the association between nutrition risk and sarcopenia in these samples, when adjusting for relevant covariates (e.g., age, sex, and use of a walker).

## 2. Materials and Methods

### 2.1. Study Design and Population

This was a secondary analysis of a cross-sectional study which involved community-dwelling older adults; the purpose of the primary study was to determine validity and reliability of SCREEN-14, a nutrition risk screening tool, in persons living with subjective cognitive impairment, their caregivers and persons living in retirement homes. Eligibility was determined through telephone screening or in-person recruitment activities. Participants needed to be 55 years of age or over; speak and read English; live in one of 44 local retirement homes for at least three months prior to participation in the study; or live in the community with at minimum, subjective cognitive impairment. Participants either had a caregiver (18+ years of age) who could provide consent on their behalf, or they demonstrated understanding of the study procedures by completing the MacArthur Competency Assessment [24]. There were no restrictions on the level of cognitive impairment among participants, and self-reported diagnosis of dementia was part of the data collection. Those living in nursing homes, receiving hospice or palliative care, or those without caregivers or failing the MacArthur Competency Assessment were excluded, as were those living outside the study region. This study was reviewed and received ethics clearance from the University of Waterloo Research Ethics Board (ORE#42827).

### 2.2. Overview of Data Collection

Data was collected over three study visits with each participant and when applicable, their caregiver. During Visit 1, participants or caregivers completed health questionnaires and the nutrition risk tool SCREEN-14 [25]. Visit 2 included review of a 24 h food intake log, the Montreal Cognitive Assessment [26], anthropometry and physical performance measures. During Visit 3, a registered dietitian completed nutritional assessment and rated the participant’s risk using the standardized Nutrition Risk Rating Score (NRRS). Participants received a nutrition consultation from the dietitian, as well as referrals to community services, if required.

Demographic variables for this analysis included gender, age group (55–64, 65–74, 75–84, ≥85 years), and living arrangement (community dwelling or retirement home). Health-related variables included Montreal Cognitive Assessment (MoCA) score (classified as impaired <26; instituted for all retirement home participants after year 1 of data collection), self-rated health (excellent, very good, good, fair, or poor), history of falls in the past 12 months (yes/no), and use of a walker (yes/no). Anthropometric measures included calf circumference (CC; described further below), height (estimated from ulna length) [27] and body weight (portable calibrated scale; without shoes in light clothing). Physical performance measures of grip strength (GS) and gait speed (GaS) were collected using standardized protocols as described further below to ensure accuracy and consistency.

### 2.3. Sarcopenia Diagnosis

Sarcopenia was diagnosed using the updated EWGSOP2 criteria following the F-A-C-S approach (see Figure 1) [11]. All participants were initially screened using the SARC-F questionnaire [28], a five-item self-report tool with scores of ≥4 indicating risk for sarcopenia. In addition, the SARC-Calf, an adapted version incorporating calf circumference, was analyzed to allow comparison of diagnostic approaches. The SARC-Calf has been proposed to improve the sensitivity of sarcopenia screening, although its performance varies across populations [29,30]. A calf circumference <34 cm for men and <33 cm for women was classified as low and assigned an additional 10 points to the SARC-F score, with total scores >11 indicating increased risk of sarcopenia [31]. Calf circumference was taken with the participant seated, feet flat on the floor and knees at approximately 90°, using a non-elastic tape measure placed around the widest part of the calf without compressing the tissue. The measurement was performed three times, and the average value was recorded.

Muscle strength was measured using a hydraulic handheld dynamometer (Jamar Hydraulic Hand Dynamometer; Performance Health, Downers Grove, IL, USA) to assess grip strength in the dominant hand unless contraindicated (e.g., injury on arm/hand). Participants were seated with their forearm supported and wrist in a neutral position and were instructed to squeeze the dynamometer as forcefully as possible. Three trials were performed with adequate rest between attempts, and the average value was recorded. Grip strength values below 27 kg for men and 16 kg for women, in combination with a positive sarcopenia risk score, were used to indicate probable sarcopenia [11]. Participants unable to complete grip strength testing were classified as meeting the low muscle strength criterion [11].

Although EWGSOP2 recommends assessing muscle quantity using techniques such as dual-energy X-ray absorptiometry (DXA), bioelectrical impedance analysis (BIA), computed tomography (CT), or magnetic resonance imaging (MRI), calf circumference may be used as a proxy when these methods are not available [11]. Calf circumference was used as a diagnostic proxy for muscle mass in this study. A calf circumference value below 31 cm was used to confirm low muscle mass and diagnose sarcopenia [11].

Finally, gait speed was assessed using a 4 m walking test in accordance with established protocols [11]. Participants were instructed to walk at their usual pace over a marked 4 m distance, with timing initiated at the first footfall after the start line and stopped at the first footfall after the finish line. The test was performed three times, with adequate rest provided between trials, and the average time was used to calculate gait speed. Walking aids (e.g., canes, walkers) were permitted and recorded where applicable. A gait speed of ≤0.8 m/s was used to indicate severe sarcopenia, and participants unable to complete the gait speed test were classified as meeting the low gait speed criterion [11]. All assessments were conducted by trained researchers using calibrated equipment.

### 2.4. Nutrition Risk

Nutrition risk was measured in two ways, SCREEN-14 and the NRRS. SCREEN-14 is a validated and reliable questionnaire for identifying nutrition risk factors among community dwelling older adults aged 55 years and older [25]. SCREEN-14 consists of 14 questions and 3 sub-questions assessing different aspects of nutritional health, including weight change, appetite, dietary intake, fluid consumption, and challenges with meal preparation and is based on the participant’s self-report of these behaviors and risk factors [25]. This tool is designed to identify individuals at nutrition risk or those with existing nutritional challenges [25]. The resulting SCREEN-14 score ranges from 0 to 64, with lower scores indicating greater nutrition risk [25]. A score of <50 indicates high risk [25]. The grocery shopping item included new response options to account for people living in retirement homes and those no longer shopping for food.

The Nutrition Risk Rating Score (NRRS) was also used to assess nutrition risk. Following Visit 3, a registered dietitian completed a standardized form and assigned each participant a score based on their nutrition risk. The NRRS incorporates body composition (body mass index, calf circumference), physical examination findings (physical exam for fat and muscle loss), weight change, physical function (gait speed), medical diagnoses, diet-related risk factors, and dietary intake based on the completed food record and analysis with ASA24-Canada-2018 (food intake software) data [32]. The NRRS has been used previously to validate SCREEN-14 and helps to standardize a dietitian’s assessment of risk [25]. Participants were assigned a score ranging from 1 to 10, with scores of 1–4 indicating low risk, 5–7 indicating moderate risk, and 8–10 indicating high nutrition risk.

To ensure the reliability of NRRS scores, each participant’s information was independently reviewed by at least one additional dietitian using blinded assessment forms, with the original score removed. A second rating was assigned based on the available participant information and documented separately. The study coordinator compared the original and second scores and identified any cases with differing scores. These cases were subsequently reviewed by a group of three dietitians, who reached consensus on the final rating for the participant. In instances where consensus could not be achieved, the higher score was assigned. These final NRRS scores were used for analysis.

### 2.5. Statistical Analysis

**Figure 2:**
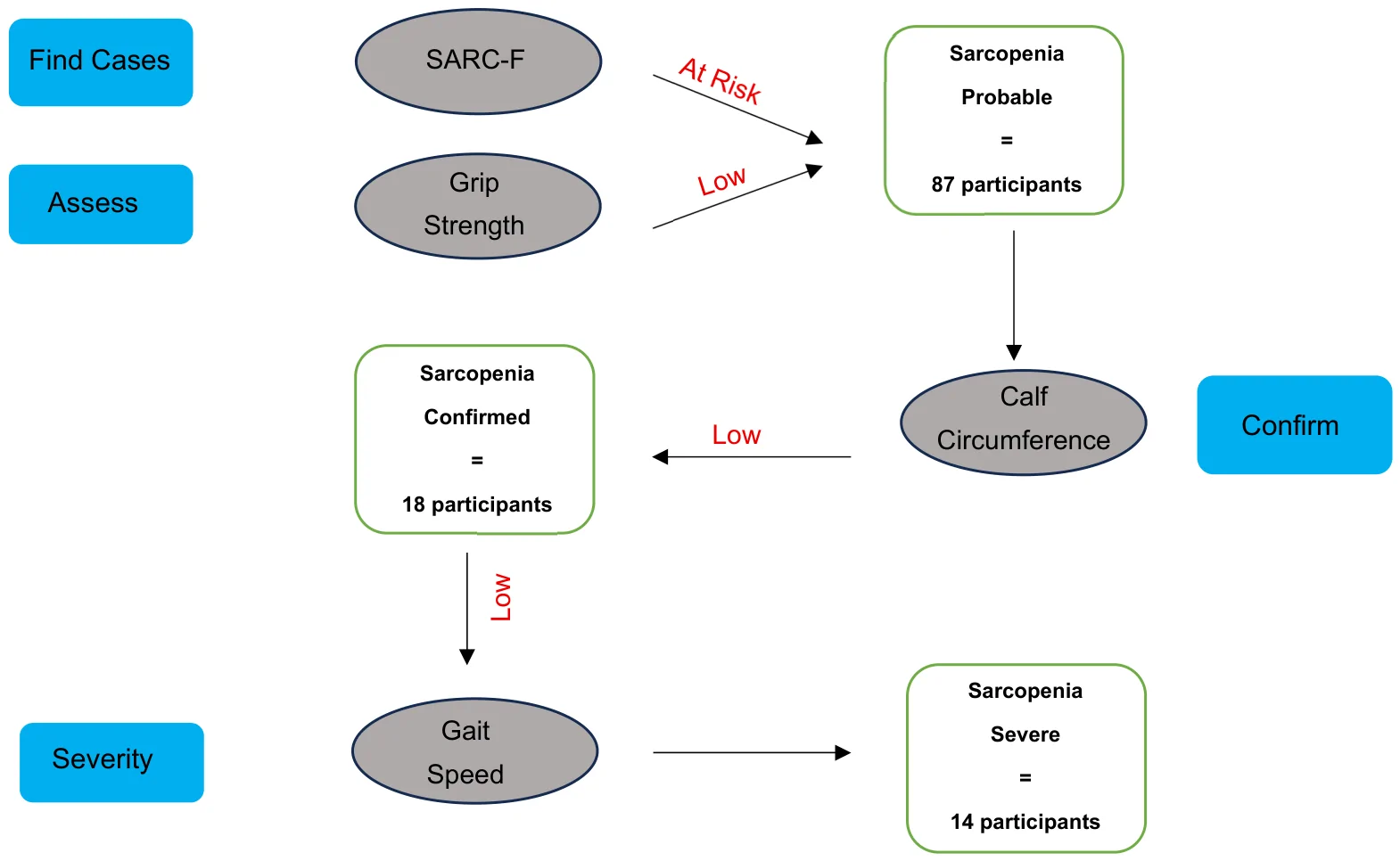
Prevalence of sarcopenia in study participants.

Data analysis was performed using SPSS (Statistics Grad Pack 31.0 Standard), with statistical significance set at *p* < 0.05. Descriptive statistics, including frequencies, percentages, means, and standard deviations were computed to summarize the demographic, health, and nutrition-related characteristics and EWGSOP2-related measures (see Table 1 and Table 2). Pearson chi-square tests were used to compare the proportion of participants meeting EWGSOP2 cut-offs and sarcopenia indicators between community and retirement groups (see Table 2). Frequencies were calculated to determine the number of participants classified as having probable, confirmed, and severe sarcopenia (see Figure 2). Post-hoc analysis was also included to demonstrate the overlap among these sarcopenia indicators as well the differing prevalence when SARC-Calf was used. Multivariable binary logistic regression analyses were conducted to examine whether sex, age, use of a walker, and nutrition risk were associated with the outcome of probable sarcopenia (yes/no; see Table 3 and Table 4). Covariates were carefully selected to ensure the models were not overfitted, as those with probable sarcopenia comprised only a subset of participants. Nutrition risk was assessed using both SCREEN-14 score (continuous, range: 0–64) and the NRRS (scale: 1–10) in two separate regression equations.

## 3. Results

The final sample consisted of 326 participants, including 153 (46.9%) community-dwelling individuals and 173 (53.1%) residents of retirement homes. The majority of participants were women (68.4%), and the most common age group was 85 years or older (44.8%). Overall, 61.7% of participants had a self-reported dementia diagnosis or a MoCA score < 26. Most participants rated their health as ‘good’ (37.7%) or ‘very good’ (29.1%). In terms of functional and health-related characteristics, 38.0% of participants reported a history of falls in the past year, and 50.6% reported using a walker. Descriptive statistics for the sample are presented in Table 1.

Descriptive statistics for EWGSOP2 measures and cut-off points are presented in Table 2. Across the total sample, 37.0% of participants were classified as at risk for sarcopenia based on SARC-F scores (≥4), 52.2% met criteria for low grip strength, 19.1% had low calf circumference, and 65.4% demonstrated slow gait speed. Mean values for SARC-F score, grip strength, calf circumference, and gait speed were 3.0 (SD = 2.5), 19.2 kg (SD = 8.6), 35.0 cm (SD = 4.6), and 0.70 m/s (SD = 0.24), respectively. A significantly greater proportion of retirement home residents were classified as at risk for sarcopenia as compared to community-dwelling participants (45.6% vs. 27.2%; χ^2^ = 11.73, *p* < 0.001). Similarly, the proportion of participants with low grip strength was significantly higher among retirement home residents (65.5% vs. 37.3%; χ^2^ = 25.81, *p* < 0.001) than those with subjective cognitive impairment in the community. Differences between groups in gait speed were also observed, with a greater proportion of retirement home residents demonstrating slow gait speed compared to community-dwelling participants (77.6% vs. 51.7%; χ^2^ = 23.88, *p* < 0.001). In contrast, there was no significant difference between groups in the proportion of participants with low calf circumference (20.3% vs. 17.6%; χ^2^ = 0.38, *p* = 0.54).

The prevalence of sarcopenia based on the EWGSOP2 diagnostic algorithm is presented in Figure 2. Overall, 87 participants (26.9%) were classified as having probable sarcopenia, defined by low grip strength following a positive screen with SARC-F. Among these participants, 18 (5.5% of the total sample) met the criteria for confirmed sarcopenia based on low calf circumference. Additionally, 14 participants (4.3% of the total sample) were identified as having severe sarcopenia, indicated by the presence of confirmed sarcopenia in combination with slow gait speed.

Results from the multivariable logistic regression model examining the association between SCREEN-14 score and probable sarcopenia are presented in Table 3. After adjusting for other variables, use of a walker was significantly associated with probable sarcopenia. Participants who reported using a walker had significantly higher odds of probable sarcopenia as compared to those who did not (OR = 11.28, 95% CI: 5.42–23.49). Additionally, females had higher odds of probable sarcopenia as compared to males (OR = 2.00, 95% CI: 1.04–3.86). Neither age nor SCREEN-14 score were associated with probable sarcopenia (age: OR = 1.02, 95% CI: 0.99–1.06; SCREEN-14 score: OR = 0.99, 95% CI: 0.95–1.02).

Results from the multivariable logistic regression model examining the association between NRRS and probable sarcopenia are presented in Table 4. After adjusting for other variables, use of a walker and NRRS were significantly associated with probable sarcopenia. Participants who reported using a walker had significantly higher odds of probable sarcopenia as compared to those who did not (OR = 6.68, 95% CI: 3.21–13.90). Additionally, higher NRRS indicating more nutrition risk as rated by a dietitian was associated with increased odds of probable sarcopenia (OR = 1.51, 95% CI: 1.21–1.88). Neither age nor sex were significantly associated with probable sarcopenia (age: OR = 1.03, 95% CI: 1.00–1.06; sex: OR = 1.89, 95% CI: 0.98–3.66).

## 4. Discussion

This study examined the prevalence of sarcopenia risk, probable and confirmed cases and its association with nutrition risk among older adults living with subjective cognitive impairment in the community and older adults living in retirement homes. Overall, sarcopenia and its related indicators were common in this sample, with over one-third of participants identified as at risk based on SARC-F, and more than half demonstrating low grip strength and slow gait speed. Consistent with the EWGSOP2 F-A-C-S framework, the prevalence decreased across diagnostic stages from at risk (37.0%), probable (26.9%), to confirmed (5.5%) and severe sarcopenia (4.3%).

In bivariate analyses, participants residing in retirement homes were at significantly greater risk for sarcopenia and demonstrated poorer muscle strength (used to assess for probable sarcopenia) and physical performance (used to determine severe sarcopenia) as compared to community dwelling individuals with subjective cognitive impairment. Interestingly, no significant differences were observed in calf circumference (i.e., the parameter used to confirm sarcopenia diagnosis) between groups, suggesting that functional measures such as grip strength and gait speed are more common components and thus more likely to rule in an older adult using the EWGSOP2 sarcopenia framework. Part of this difference in prevalence in these two settings may be a result of age differences in the samples, with almost two-thirds of retirement home participants being older than 85 years.

The observed prevalence of sarcopenia in this study is consistent with global estimates. A systematic review and meta-analysis [33] reported sarcopenia prevalence ranging from 10% to 27%, with severe sarcopenia between 2% and 9%. In the present study, 26.9% of participants were classified as having probable sarcopenia and 4.3% of participants were classified as having severe sarcopenia. Similarly, another study [34] demonstrated that prevalence estimates are generally lower when using EWGSOP2 as compared to EWGSOP, with reported ranges of 3.2% to 26.3%. The prevalence of confirmed sarcopenia observed in the current study (5.5%) is within this range but on the low end of these estimates. These prevalence values emphasize the need for regular sarcopenia screening.

A key distinction between EWGSOP and EWGSOP2 lies in the emphasis on muscle strength in EWGSOP2, which is given more weight than muscle mass, the primary factor in EWGSOP [5,11,35]. This shift may explain the lower prevalence of confirmed sarcopenia observed, as individuals with reduced physical performance (e.g., slow gait speed) but preserved muscle strength do not meet diagnostic criteria. In this study, a substantial proportion of participants demonstrated slow gait speed (n = 210, 65.4%); however, many did not meet the full EWGSOP2 diagnostic criteria for sarcopenia. This analysis suggests that muscle mass determination, which was made with calf circumference after ‘probable sarcopenia’ (i.e., low grip strength) and before severe sarcopenia determination (i.e., slow gait speed) is the limiting feature of EWGSOP2 that drove the low prevalence in this study. This comparison illustrates how the application of the full EWGSOP2 diagnostic algorithm reduces case identification compared to the use of individual indicators. Muscle mass determination through calf-circumference needs to be carefully considered in future diagnostic frameworks.

Post-hoc analyses highlighted potential limitations of SARC-F as a standalone screening tool. In this sample, 210 participants demonstrated slow gait speed (≤0.8 m/s); however, 101 of these individuals (49.0%) were not classified as at risk based on SARC-F. This suggests that nearly half of participants with impaired physical performance may be missed when relying solely on SARC-F, indicating potential under-detection of sarcopenia-related functional impairment. This finding is consistent with previous literature suggesting that SARC-F has good specificity but limited sensitivity [10,11,29,30].

While modifications such as SARC-Calf have been proposed to improve case identification, applying a SARC-Calf threshold (>11) in combination with slow gait speed in this study identified fewer individuals in post-hoc analyses with probable sarcopenia (n = 55) as compared to the EWGSOP2-based approach using SARC-F and grip strength (n = 87). Using calf circumference demonstrates only low-to-moderate agreement with reference standards and variable diagnostic accuracy across populations, potentially contributing to under- or over-identification of sarcopenia [36]. Evidence suggests that the diagnostic performance of SARC-Calf is highly dependent on the choice of calf circumference cut-off values, with different thresholds potentially leading to underestimation of sarcopenia, particularly across different sexes and population [30]. Furthermore, as calf circumference can be influenced by factors such as adiposity or edema, it may mask low muscle mass in some individuals, further limiting its reliability as a standalone proxy measure [30].

The concept of muscle quality introduced in EWGSOP2 further complicates the classification [11]. The assessment of muscle quality is relatively new and requires specialized techniques such as CT scans or MRI, which were not included in our study and are not typically available in community settings [11]. Additionally, more recently, the Global Leadership Initiative in Sarcopenia (GLIS) has moved away from including muscle quality as a diagnostic construct, instead proposing muscle-specific strength (muscle strength relative to muscle size) as a core component of the conceptual definition of sarcopenia, alongside muscle mass and muscle strength, while recognizing physical performance as an outcome rather than a defining component [2]. Consequently, our findings should be interpreted within the EWGSOP2 framework, recognizing that the absence of direct measures of muscle quality or muscle-specific strength may have limited the ability to identify individuals with impaired muscle function despite preserved muscle mass or strength.

A key finding of this study was the differential performance of nutrition risk measures. While SCREEN-14 scores were not significantly associated with probable sarcopenia, higher NRRS was significantly associated with increased odds of probable sarcopenia. This may suggest that clinician-informed assessments, which incorporate clinical judgment and multiple domains of nutritional and functional status, may be more closely aligned with sarcopenia risk than self-reported screening tools. However, components of the NRRS include handgrip strength, calf-circumference, walking speed as well as the SARC-F scores. Thus, there is overlap in the EWGSOP2 framework and the NRRS standardized assessment, and an association is to be expected. An abbreviated version of SCREEN has demonstrated predictive validity with performance measures [37]. The lack of association in this study may be a result of the low prevalence of probable sarcopenia when following the EWGSOP2 framework or that SCREEN-14 focuses on upstream determinants of dietary intake rather than functional impairment [38].

Across both regression models, use of a walker was strongly associated with probable sarcopenia, highlighting the importance of mobility limitations as a key correlate of sarcopenia risk. In contrast, age was not significantly associated with probable sarcopenia.

### 4.1. Strengths

The use of EWGSOP2 criteria for diagnosing sarcopenia is a major strength of this study, as these guidelines are internationally recognized for defining sarcopenia based on physical performance, muscle strength, and muscle quantity/quality [11]. It is well-recognized that sarcopenia is associated with cognitive impairment [39]. Thus, we were justified in using this secondary data where two-thirds of the sample had cognitive impairment to explore associations between nutrition risk and probable sarcopenia. The inclusion of retirement home residents is a novel aspect of this work, as this population continues to grow in Canada [17,18] and elsewhere. Use of both self-reported and clinician-rated nutrition risk tools also allowed for a comparison of assessment approaches.

### 4.2. Limitations

The limitations of this study must also be acknowledged. The sample was predominantly female (68.4%), thus, future studies should aim to recruit a more balanced sex distribution to improve the applicability of the findings. Participants also were as young as 55 years of age, as this was the minimum age for eligibility in the main validation study. The two groups significantly varied in age, with retirement participants being older. It is known that retirement home residents are similar to long-term care residents when making comparisons with demographic data and some health parameters [17]. To recruit community-living participants, presentations at booths and health fairs were key sources. This resulted in a younger and more mobile or active demographic; recruitment from geriatric clinics and memory clinics was very challenging and yielded few participants. The requirement for three study visits also limited recruitment, especially of frail older adults. Additionally, calf circumference was used as a measure of muscle mass. While this method is widely accessible and inexpensive, it is less accurate than the more precise techniques recommended by EWGSOP2, such DXA, BIA, CT, or MRI [11]. Further, for modeling probable sarcopenia, we were limited in the number of covariates that could be considered, due to the relatively small proportion in our sample with probable sarcopenia as per the EWGSOP2 criteria. Although bivariate analyses suggest significant differences in sarcopenia indicators between retirement home and community participants (Table 2), we elected to focus on the NRRS and demographic factors in the regression model. Future research should include larger samples for regression analyses to explore potential differences in prevalence of probable, confirmed, and severe sarcopenia with living location. Finally, SCREEN-14 was being tested for validity in this cross-sectional study and may not fully represent nutrition risk of older adults with cognitive impairment or living in retirement residences. Further, due to the cross-sectional nature of the design, no conclusions should be drawn from associations identified between the NRRS, walker use and probable sarcopenia.

## 5. Conclusions

This study highlights the prevalence of sarcopenia risk (37%) among community dwelling older adults, though the levels of probable (27%), confirmed (5.5%), and severe sarcopenia (4.3%) as per the EWGSOP2 criteria were relatively low. Retirement home residents were found to be at significantly higher risk for sarcopenia than older adults living in the community with subjective cognitive impairment. These findings demonstrate the heightened vulnerability of retirement home residents and suggests that sarcopenia screening should be prioritized in these congregate-living settings. Nutrition risk as assessed by a dietitian was associated with this risk, as was use of a walker. Given the widespread prevalence of sarcopenia risk, our study suggests that it is important to screen for sarcopenia. The SARC-F tool missed many individuals who had slow gait and low hand-grip strength in this study. The Global Leadership Initiative in Sarcopenia has developed consensus on the definition of sarcopenia [2] and will next be considering how to measure sarcopenia in diverse populations. As these recommendations evolve, future work should evaluate how emerging GLIS-based approaches perform across diverse populations, including community dwelling older adults with cognitive impairment or dementia diagnosis and retirement home residents.

## Figures and Tables

**Table 1:** Participant demographics.

	Total (n = 326)	%	Community (n = 153, 46.9%)	%	Retirement Home (n = 173, 53.1%)	%
**Gender**						
Man	102	31.3	55	35.9	47	27.2
Woman	223	68.4	97	63.4	126	72.8
Other	1	0.3	1	0.7		
**Age**						
55–64 years	23	7.1	19	12.4	4	2.3
65–74 years	45	13.8	37	24.2	8	4.6
75–84 years	112	34.4	62	40.5	50	28.9
85+ years	146	44.8	35	22.9	111	64.2
**Dementia Diagnosis or MoCA < 26**						
Yes	201	61.7	104	68.0	97	56.1
No	125	38.3	49	32.0	76	43.9
**Self-Rated Health**						
Excellent	23	7.1	9	5.9	14	8.1
Very Good	95	29.1	37	24.2	58	33.5
Good	123	37.7	61	39.9	62	35.8
Fair	71	21.8	38	24.8	33	19.1
Poor	13	4.0	8	5.2	5	2.9
Prefer Not to Say	1	0.3			1	0.6
**History of Falls**						
Yes	124	38.0	54	35.3	70	40.5
No	201	61.7	99	64.7	102	59.0
Prefer not to say	1	0.3			1	0.6
**Use of Walker**						
Yes	165	50.6	45	29.4	120	69.4
No	161	49.4	108	70.6	53	30.6
**SCREEN Visit 1**						
Interview administered	320	98.2	152	99.3	168	97.1
Self-administered	6	1.8	1	0.7	5	2.9
**SCREEN-14 score, median (IQR)**	48 (43–53)		47 (41.3–51.8)		50 (45–54)	
**NRRS, median (IQR)**	7 (5-8)		7 (5-8)		7 (6-8)	

Note. MoCA = Montreal Cognitive Assessment. MoCA was not completed in all retirement home participants. NRRS = Nutrition risk rating scale.

**Table 2:** EWGSOP2 measures and cut-off points in sample from community and retirement homes.

	All				Retirement				Community			
	%	n	Mean	SD	%	n	Mean	SD	%	n	Mean	SD
**Find:**												
SARC-F Score		322	3.0	2.5		171	3.4	2.6		151	2.5	2.4
At Risk (≥4) ***	**37.0**	119			**45.6**	78			**27.2**	41		
**Assess:**												
Grip Strength (kg)		324	19.2	8.6		171	16.6	6.9		153	22.2	9.3
Low (<27 kg male & <16 kg female) ***	**52.2**	169			**65.5**	112			**37.3**	57		
**Confirm: Calf Circumference**												
Calf Circumference (cm)		325	35.0	4.6		172	34.7	4.5		153	35.4	4.6
Low (<31 cm)	**19.1**	62			**20.3**	35			**17.6**	27		
**Severity:**												
Gait Speed (m/s)		321	0.70	0.24		170	0.63	0.19		151	0.78	0.26
Slow (≤0.8 m/s) ***	**65.4**	210			**77.6**	132			**51.7**	78		

Note. Sample sizes (n) vary across measures because of missing data. *** *p* < 0.001 for differences between retirement and community groups.

**Table 3:** Odds ratios for the binary logistic regression testing the association between age, sex, use of walker, and SCREEN-14 score with probable sarcopenia.

Effect	Odds Ratio (Exp (B))	95% Confidence Limits	
Age (Per year increase)	1.02	0.99	1.06
**Sex (Female vs. Male)**	**2.00**	**1.04**	**3.86**
**Use of Walker (Yes vs. No)**	**11.28**	**5.42**	**23.49**
SCREEN-14 score (Per 1-point increase)	0.99	0.95	1.02

Note. Bolded values indicate statistical significance (*p* < 0.05). Reference categories: Male for Sex; No for Use of Walker.

**Table 4:** Odds ratios for the binary logistic regression testing the association between age, sex, use of walker, and NRRS with probable sarcopenia.

Effect	Odds Ratio (Exp (B))	95% Confidence Limits	
Age (Per year increase)	1.03	1.00	1.06
Sex (Female vs. Male)	1.89	0.98	3.66
**Use of Walker (Yes vs. No)**	**6.68**	**3.21**	**13.90**
**NRRS**	**1.51**	**1.21**	**1.88**

Note. Bolded values indicate statistical significance (*p* < 0.05). Reference categories: Male for Sex; No for Use of Walker.

## Data Availability

The datasets presented in this article are not readily available because consent for sharing beyond the researchers involved in this study was not requested of participants. Summarized data are available from H.H.K.
